# Supplementary material for: GCH1 p.Ser80Asn Confers Risk for Parkinson’s Disease in East Asian Populations
Source: medRxiv. 2026 Jun 22:2026.06.11.26354827. Preprint. [Version 1] doi: 10.64898/2026.06.11.26354827 (PMC13321183; doi:10.64898/2026.06.11.26354827)
Supplement: Supplement 1 [file media-1.pdf]

Supplementary Materials

GCH1 p.Ser80Asn Confers Risk for Parkinson's Disease in East Asian Populations

Yi Wen Tay[1,2,a*], Andrew Leslie Lee[2,a], Jie Ping Schee[2], Chin Hsien Lin[3], Eng King Tan[4,5], Jung Hwan Shin[6], Pin-Shiuan Chen[7], Sung-Pin Fan[3], Cheng-Hsuan Li[3], Ebonne Ng Yu Lin[5], Han Joon Kim[6], Beomseok Jeon[6], Sulev Koks[,8,9], Kin Ying Mok[10,11], Yi Ting Lim[12], Mohd Salahuddin Kamaruddin[12], Tzi Shin Toh[2], Hans Xing Ding[13], Anis Nadhirah Khairul Anuar[13], Norlisah Ramli[14], Ignacio Juan Keller Sarmiento[15], Maria Teresa Periñan[16,17], Zih-Hua Fang[18], Lara M Lange[19,20], Kishore R. Kumar[21,22,23], Soraya Bardien[24,25], Joanne Trinh[20], Enza Maria Valente[26,27], SG10K_Health Consortium, the Global Parkinson's Genetics Program (GP2), Peter Heutink[20], Katja Lohmann[20], Christine Klein[20], Niccolò E Mencacci[15], Shen-Yang Lim[2], Azlina Ahmad-Annuar[1], Ai Huey Tan[2*]

[1]Department of Biomedical Science, Faculty of Medicine, University of Malaya, Kuala Lumpur, Malaysia
[2]Division of Neurology, Department of Medicine, Faculty of Medicine, University of Malaya, Kuala Lumpur, Malaysia
[3]Department of Neurology, National Taiwan University Hospital Taipei, Taipei, Taiwan
[4]Duke-National University of Singapore Medical School, Singapore
[5]Department of Neurology, National Neuroscience Institute, Singapore General Hospital, Singapore
[6]Department of Neurology, Seoul National University Hospital, College of Medicine, Seoul National University, Seoul, Republic of Korea
[7]Department of Neurology, National Taiwan University Hospital Bei-Hu branch, Taipei, Taiwan
[8]Personalised Medicine Centre, Health Futures Institute, Murdoch University, Perth, Australia
[9]Perron Institute for Neurological and Translational Science, Perth, Australia
[10]The Hong Kong University of Science and Technology, Clear Water Bay, Kowloon, Hong Kong SAR, China
[11]Hong Kong Center for Neurodegenerative Diseases, Hong Kong Science Park, Shatin, HKSAR, China
[12]Department of Biomedical Imaging, Faculty of Medicine, University of Malaya, Kuala Lumpur, Malaysia
[13]Department of Physiology, Faculty of Medicine, University of Malaya, Kuala Lumpur, Malaysia
[14]Department of Radiology, Subang Jaya Medical Centre, Selangor, Malaysia
[15]Ken and Ruth Davee Department of Neurology and Simpson Querrey Center for Neurogenetics, Northwestern University, Feinberg School of Medicine, Chicago, Illinois, USA
[16]Centre for Preventive Neurology, Wolfson Institute of Population Health, Queen Mary University of London, London, United Kingdom
[17]Unidad de Trastornos del Movimiento, Servicio de Neurología y Neurofisiología Clínica, Instituto de Biomedicina de Sevilla, Hospital Universitario Virgen del Rocío/Consejo Superior de Investigaciones Científicas (CSIC)/Universidad de Sevilla, Seville, Spain
[18]DataTecnica LLC, Washington, DC 20037, USA

[19]Laboratory of Neurogenetics, National Institute on Aging, National Institutes of Health, Bethesda, MD, USA
[20]Institute of Neurogenetics, University of Luebeck, Luebeck, Germany
[21]Translational Neurogenomics Group, ANZAC Research Institute, Sydney Local Health District and Faculty of Medicine and Health, The University of Sydney, Sydney, New South Wales, Australia
[22]Garvan Institute of Medical Research, Sydney, NSW 2010, Australia
[23]School of Clinical Medicine, UNSW Medicine & Health, University of New South Wales, Kensington, NSW, Australia
[24]Division of Molecular Biology and Human Genetics, Department of Biomedical Sciences, Faculty of Medicine and Health Sciences, Stellenbosch University, Cape Town, South Africa
[25]South African Medical Research Council/Stellenbosch University Genomics of Brain Disorders Research Unit, Cape Town, South Africa
[26] Neurogenetics Research Center, IRCCS Mondino Foundation, 27100 Pavia, Italy
[27]Department of Molecular Medicine, University of Pavia, 27100 Pavia, Italy

[a]Joint first author; *Corresponding author

Correspondence to:
Ai Huey, Tan
Division of Neurology, Department of Medicine, Faculty of Medicine, University of Malaya, Kuala Lumpur, Malaysia
Email: aihuey.tan@gmail.com

Yi Wen, Tay
Department of Biomedical Science, Faculty of Medicine, University of Malaya, Kuala Lumpur, Malaysia
Email: tayyiwen96@gmail.com

This file includes:

1. **Supplementary Methods …………………………………………….………….. 3**
2. **Supplementary Results ……………………………………….……………….. 5**
3. **Supplementary Tables …….…………………………………………………....7**

Supplementary Methods

Genetic screening in proband and family

Peripheral blood (10mL) was collected from each subject. DNA was extracted using the FavorPrep™ Genomic DNA Extraction Maxi Kit (Favorgen, Taiwan) according to the manufacturer's protocol. The proband was screened via whole genome sequencing (WGS) via Global Parkinson's Genetics Program (GP2). Single nucleotide polymorphisms (SNPs), copy number variations (CNVs) and repeat expansions in known PD-related genes (Supplementary Table 2) were analysed. Candidate variants were validated via Sanger sequencing in the proband and his family members.

Additionally, the proband was screened via the SALSA® multiplex ligation-dependent probe amplification (MLPA) Probemix P051 kit (MRC Holland, Amsterdam, the Netherlands). (MLPA) assay for CNVs in known PD genes (*PRKN*, *PINK1*, *DJ-1*, *SNCA*, and *ATP13A2* [exons 2 and 9]). The data were analysed using the Coffalyzer software (https://www.mrcholland.com/technology/software/coffalyser-net).

Carrier frequency screening, association and haplotype analyses

In the GP2 WGS R11 datasets ($n=22{,}372_{PD}$; $n=3{,}578_{\text{other neurological disorders}}$; $n=8{,}826_{\text{Control}}$), populations across 11 ancestries were analyzed, including African American (AAC), African (AFR), Ashkenazi Jewish (AJ), Admixed American/Latin American (AMR), Complex Admixture History (CAH), Central Asian (CAS), East Asian (EAS), European (EUR), Middle Eastern (MDE), Finnish (FIN) and South Asian (SAS) (Supplementary Table 2).

The PLINK binary files of WGS data were processed using the standard quality control procedures of the GenoTools pipeline.[1] Samples with call rate >0.95, quality >20, read depth >5, and heterozygous allele balance between 0.25 and 0.75 were retained. Duplicated samples (kinship coefficient >0.354) and those with sex mismatches were removed. Ancestry prediction was performed via GenoTools[1] using the default reference panels from the 1000 Genomes Project, the Human Genome Diversity Project, and Ashkenazi Jewish datasets. Related individuals with second-degree or closer relatedness (kinship coefficient >0.0884) were excluded, leaving only the index cases for downstream analysis.

The genomic coordinates of 1kbp upstream and downstream *GCH1* p.Ser80Asn variant were obtained from the Ensembl database (hg38: chr14:54,901,425 - 54,903,425; https://www.ensembl.org). Variants within the region were extracted using PLINKv2.0, followed by annotation with ANNOVAR using reference datasets including refGene, dbNSFP v4.7a, ClinVar and gnomAD v4.1.1.

Additionally, GP2 WGS EAS cohort ($n=2{,}757_{PD}$ and $n=1{,}020_{\text{controls}}$) and independent EAS cohorts, including the EAS whole exome sequencing study (EAS-WES)[2] ($n=1{,}955_{PD}$ and $5{,}512_{\text{controls}}$), the Singaporean whole genome-based SG10K_Health project[3] ($n=9{,}770_{\text{controls}}$), and EAS controls from gnomAD (v4.1.0) ($n=22{,}431_{\text{controls}}$) were meta-analyzed to validate the findings. A two-tailed Fisher's exact test was used to compare allele frequencies of the *GCH1*

p.Ser80Asn variant between cases and controls, and to estimate odds ratios (OR) with 95% confidence intervals (CI).

To determine whether the *GCH1* p.Ser80Asn variant carriers harbored a shared common haplotype, we phased chromosome 14 WGS data using Beagle v5.4[4] and searched for shared identical-by-descent (IBD) segments of length ≥2 cM among carriers using the hap-ibd function.[5] In addition, haplotypes surrounding the *GCH1* p.Ser80Asn variant were assessed using five flanking microsatellite markers (D14S991, D14S1057, D14S276, D14S52, and D14Skh3), previously described[6], spanning approximately 1.03 Mbp, with inter-marker distances ranging from 32 to 143 kbp. Genotyping was performed in nine Malaysian and Taiwanese carriers (Subjects 5-11, Subject 13 and his mother), Subject 13's unaffected father and brother (I:1 and II:1; Figure 1A), as well as five EAS controls. Moreover, SNP markers from GP2 WGS data of the carriers and 50 EAS controls were extracted across the same 1 Mbp region and used to supplement the microsatellite-based haplotype analysis and facilitate fine mapping. SNPs flanking the *GCH1* p.Ser80Asn variant were manually inspected, and the nearest upstream and downstream variants consistently observed in the non-reference state (heterozygous or homozygous alternate) among carriers were considered informative markers for defining the putative shared haplotype.

Literature search for carriers of the *GCH1* p.Ser80Asn variant

A PubMed search was conducted using the terms ("GCH1" OR "GTP cyclohydrolase 1") AND (dystonia OR "dopa-responsive dystonia" OR "DRD" OR "parkinson" OR "Parkinson's disease"), with no language restrictions for publications until 31st December 2025. All published cases carrying the *GCH1* p.Ser80Asn variant were extracted and summarized. Collaborators were contacted for clinical phenotyping data for identified *GCH1* p.Ser80Asn cases within the GP2 cohort.

Supplementary Results

Clinical, neuroimaging and genetic findings of the proband and his family

Case description of the proband

The proband is a Chinese male (II:2, Figure 1A) from a non-consanguineous family who presented at age ranging 40-50 with progressive slowness in movements and gait impairment, without diurnal variation. On examination, he had bilateral moderate rigidity and bradykinesia, worse in the right upper and lower limbs. His gait was impaired with mild right foot dystonia characterised by right ankle inversion and toe extension, difficulty in initiation, short stride length and reduced arm swing. His eye movements and speech were normal. There were no tremor, other movement disorders, or pyramidal signs. His parkinsonism progressively worsened over several years, requiring increasing doses (LEDD: 950mg/day) of antiparkinsonian medications. By seven years post-diagnosis (at age ranging 50-60), he developed motor fluctuations ("wearing off" phenomenon lasting approximately three hours a day) and exhibited levodopa-induced dyskinesias.

At the most recent follow-up, the proband retained independence in activities of daily living but was unable to continue working as a driver. There was a progressive increase in disease severity, with his Clinical Impression of Severity Index for PD (CISI-PD) rising from 5 in 2019 to 12 in 2026. Between 2024 and 2026, his total MDS-UPDRS score increased slightly from 52 to 54, with a notable increase in non-motor burden (Part I: 3 to 8), and stable ON-state motor scores (Part III: 32 to 34). Current therapy consisted of levodopa–benserazide, piribedil, and selegiline (levodopa equivalent daily dosage [LEDD] 950 mg/day at 10 years of disease duration). Cognitive screening revealed mild impairment (Montreal Cognitive Assessment score 19/30; 9 years of formal education). Non-motor symptoms included REM sleep behaviour disorder (RBD), constipation, orthostatic hypotension, and pain. His medical history included essential hypertension and severe obstructive sleep apnoea (OSA) managed with continuous positive airway pressure (CPAP).

DaT-SPECT and 99mTc-TRODAT-1 SPECT/MRI fusion images (Figure 1B, v–vii) confirmed advanced nigrostriatal neurodegeneration with marked bilateral involvement of the putamen. This deficit was notably more pronounced on the left side, correlating with the patient's predominant right-sided motor findings. Axial SWI sequence images (Figure 1B, viii) revealed a loss of the left "swallow tail" sign and nigrosome-1 hyperintensity.

Case description of the proband's mother

His mother (I:2, Figure 1A) was incidentally found to have mild parkinsonism at age ranging 70-80 in 2024, characterized by mild bradykinesia in all limbs and slight rigidity on reinforcement, more notable on the right side. Gait was slow but with good stride length and arm swing. She did not have tremor, dystonia, other movement disorders, or pyramidal signs. Longitudinal assessment from 2024 to 2026 revealed progressive decline, with her total MDS-UPDRS score increasing from 32 to 49, primarily driven by worsening motor examination findings (Part 3: 23 to 34). By 2026, her CISI-PD was 5, and cognitive screening showed mild impairment (MoCA 21/30; six years of formal education). Over the two-year follow-up, she continued to be

fully independent and employed in manual work. She was treated with low-dose levodopa–benserazide (LEDD 100 mg/day at 2 years of disease duration).

Motor and non-motor features of both proband and his mildly affected mother are summarised in Table 1 with accompanying videos in the Supplementary Materials. DaT-SPECT and 99mTc-TRODAT-1 SPECT/MRI fusion images (Figure 1B, i–iii) demonstrated nigrostriatal neurodegeneration characterized by mild, primarily unilateral loss. In contrast to the functional imaging, structural assessment via axial SWI sequence (Figure 1B, iv) showed a preserved bilateral "swallow tail" sign (white arrows).

Haplotype analyses

As the variant was exclusively observed in individuals of EAS ancestry within the GP2 WGS cohort, haplotype analyses were performed to evaluate whether carriers shared a common ancestral haplotype. However, no shared disease-associated haplotype was detected among the 10 *GCH1* p.Ser80Asn carriers following Beagle phasing. Consistent with this result, neither microsatellite marker analysis nor inspection of flanking SNP genotype patterns across the same ~1 Mbp region identified a common disease-associated haplotype among the *GCH1* p.Ser80Asn carriers (Supplementary Table 3). A schematic diagram to illustrate the segregation of microsatellite and SNP marker alleles in the Malaysian Chinese family was shown in Supplementary Figure 1.

Supplementary Figure 1: Haplotype analyses surrounding the *GCH1* p.Ser80Asn variant in a Malaysian Chinese family using microsatellite and SNP markers.

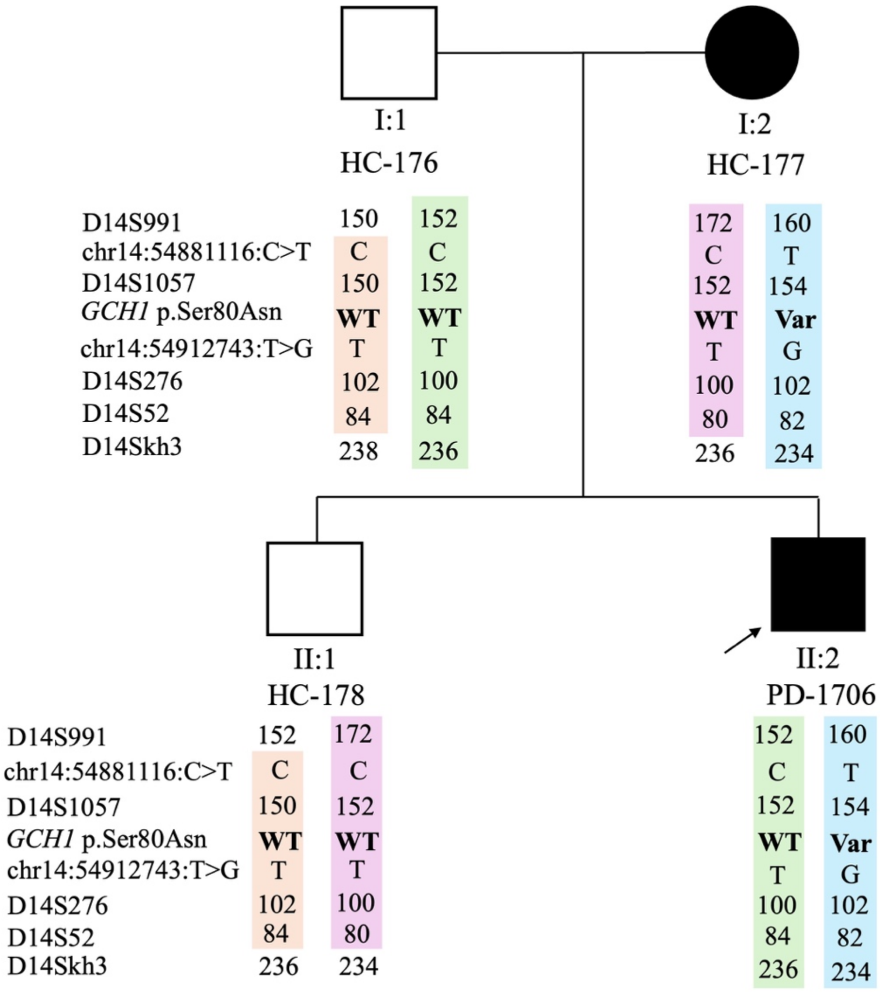

Schematic representation of the segregation of microsatellite and SNP marker alleles flanking the *GCH1* p.Ser80Asn variant within the Malaysian Chinese family. Var=Variant; WT=Wildtype.

Supplementary Tables

Supplementary Table 1: Demographics of the multi-ancestral GP2 R11 cohorts with whole genome sequencing data.

Genetic ancestry	Number of subjects			*GCH1* p.Ser80Asn variant carrier, *n*
	PD, *n* [1]	Patients with other neurological disorders, *n* [2]	Control, *n* [3]	
AAC	245	6	157	0
AFR	1,495	33	2,063	0
AJ	1,190	52	234	0
AMR	907	8	300	0
CAH	234	25	117	0
CAS	684	125	954	0
EAS	2,757	474	1,020	10<br>(9 PD and 1 control)
EUR	13,416	2,761	2,678	0
FIN	20	5	4	0
MDE	945	26	967	0
SAS	479	63	332	0
**Total**	**22,372**	**3,578**	**8,826**	**10**

AAC=African American; AFR=African; AJ=Ashkenazi Jewish; AMR=Admixed American/Latin American; CAH=Complex Admixture History; CAS=Central Asian; EAS=East Asian; EUR=European; FIN=Finnish; MDE=Middle Eastern; *n*=Number; SAS=South Asian.

[1] The "PD" category includes individuals with PD from i) unselected PD case/control cohorts, ii) monogenic recruitment, i.e., targeted recruitment of individuals in which a monogenic cause of disease is more likely based on a young age at onset <50 years and/or a positive family history of

PD), and iii) genetically enriched cohorts, i.e., targeted recruitment of carriers of pathogenic variants in *GBA1*, *LRRK2*, or *SNCA*.

[2] The “Other neurological disorders” category summarizes individuals with phenotypes other than classical PD, including atypical forms of parkinsonism (e.g., multisystem atrophy, progressive supranuclear palsy, corticobasal syndrome, and dementia with lewy bodies) and other neurodegenerative phenotypes (e.g., Alzheimer’s disease, mild cognitive impairment, other forms of dementia).

[3] The “control” category summarizes all individuals without PD or other phenotypes listed above, including healthy controls, population cohorts, and healthy family members of individuals with PD or parkinsonism.

Supplementary Table 2: PD and neurological disorder-related genes screened in the proband of the Malaysian Chinese family via whole genome sequencing.

**A. PD and neurological disorder-related genes screened for single nucleotide polymorphism and copy number variations**				
AFG3L2	*EPM2A*	*NR4A2*	*QDPR*	*SYNJ1*
ATP13A2	*FA2H*	*OPA1*	*RAB39B*	*TAF1*
ATP1A3	*FBXO7*	*OPA3*	*SCP2*	*TBK1*
ATP6AP2	*FTL*	*PANK2*	*SLC20A2*	*TH*
ATP7B	*GBA*	*PARK7*	*SLC25A4*	*TUBB4A*
C19orf12	*GCH1*	*PDE8B*	*SLC25A46*	*TWNK*
CHCHD10	*GLB1*	*PDGFB*	*SLC30A10*	*VAC14*
CHCHD2	*GRN*	*PDGFRB*	*SLC39A14*	*VCP*
COASY	*JAM2*	*PINK1*	*SLC6A3*	*VPS13A*
CP	*LRRK2*	*PLA2G6*	*SMPD1*	*VPS13C*
CSF1R	*LYST*	*POLG*	*SNCA*	*VPS35*
DCAF17	*MAPT*	*PRKN*	*SPG11*	*WDR45*
DCTN1	*MYORG*	*PRKRA*	*SPG7*	*XPR1*
DNAJC12	*NPC1*	*PTRHD1*	*SPR*	*ZFYVE26*
DNAJC6	*NPC2*	*PTS*	*SQSTM1*	
**B. PD and neurological disorder-related genes screened for repeat expansions**				
AFF2	*ATXN7*	*DMPK*	*NIPA1*	*RFC1-AAGGG*
AR	*AYXN8OS*	*FGF14*	*NOP56*	*RFC1-ACAGG*
ARX_1	*BEAN1*	*FMR1*	*NOTCH2NLC*	*SAMD12*
ARX_2	*C9ORF72*	*FXN*	*NUTM2B-AS1*	*SOX3*
ATN1	*CACNA1A*	*GIPC1*	*PABPN1*	*STARD7*
ATXN1	*CBL*	*GLS*	*PHOX2B*	*TBP*
ATXN10	*CNBP*	*HTT*	*PPP2R2B*	*TNRC6A*
ATXN2	*DAB1*	*JPH3*	*PRDM12*	*YEATS2*
ATXN3	*DIP2B*	*MARCHF6*	*RAPGEF2*	*ZIC2*

Supplementary Table 3: Haplotypes analyses surrounding the *GCH1* p.Ser80Asn variant in East Asian carriers and controls using microsatellite and SNP markers.

**Subject**		**5**	**6**	**7**	**8**	**9**	**10**	**11**	**12**	**13 - Proband**	**13's mother**	**C1**	**13's father**	**13's brother**	**C2**	**C3**	**C4**	**C5**	**C6**	**C7**	**C8**
**Microsatellite or SNP markers**	**Chr position (hg38)**																				
D14S991	54757307	144/150	144/152	144/152	144/150	144/152	132/132	152/150	NA	152/160	172/160	NA	150/152	152/172	150/160	134/150	156/160	152/160	134/134	NA	NA
chr14:54881116:C>T	54881116	C/T	C/T	T/T	C/T	C/T	C/T	C/T	C/T	C/T	C/T	C/T	C/C	C/C	C/C	C/C	C/C	C/C	C/C	C/T	T/T
D14S1057	54900756	152/154	152/154	152/154	152/154	166/154	162/154	152/154	NA	152/154	152/154	NA	150/152	150/152	158/162	152/150	152/156	148/152	148/164	NA	NA
GCH1 p.Ser80Asn	54902425	WT/Var											WT/WT								
chr14:54912743:T>G	54912743	T/G	T/G	T/G	T/G	T/G	T/G	T/G	T/G	T/G	T/G	T/G	T/T	T/T	T/T	T/T	T/T	T/T	T/T	T/G	T/G
D14S276	55216298	94/102	94/102	94/106	100/102	94/94	94/94	94/102	NA	100/102	100/102	NA	102/100	102/100	104/94	102/106	102/102	100/102	100/106	NA	NA
D14S52	55758620	84/86	84/86	82/86	74/86	82/86	80/82	72/82	NA	84/82	80/82	NA	84/84	84/80	74/74	82/84	80/80	86/88	86/88	NA	NA
D14Skh3	55790598	236/234	226/238	230/238	230/238	228/230	230/236	228/230	NA	236/234	236/234	NA	238/236	236/234	228/230	230/236	234/242	228/230	236/234	NA	NA

Genotype patterns of the flanking microsatellite and informative SNP markers surrounding the GCH1 p.Ser80Asn variant in carriers and East Asian (EAS) controls are shown. Two flanking SNPs, chr14:54881116:C>T (upstream) and chr14:54912743:T>G (downstream), consistently observed in the non-reference state (heterozygous or homozygous alternate) among carriers were selected as informative markers. C=Control; SNP=Single nucleotide polymorphism; Var=Variant; WT=Wildtype.

References

1. Vitale D, Koretsky MJ, Kuznetsov N, et al. GenoTools: an open-source Python package for efficient genotype data quality control and analysis. G3 (Bethesda) 2025;15(1).
2. Chew EG, Liu Z, Li Z, et al. Exome sequencing in Asian populations identifies low-frequency and rare coding variation influencing Parkinson's disease risk. Nat Aging 2025;5(2):205-18.
3. Wu D, Dou J, Chai X, et al. Large-Scale Whole-Genome Sequencing of Three Diverse Asian Populations in Singapore. Cell 2019;179(3):736-49 e15.
4. Browning BL, Tian X, Zhou Y, Browning SR. Fast two-stage phasing of large-scale sequence data. Am J Hum Genet 2021;108(10):1880-90.
5. Zhou Y, Browning SR, Browning BL. A Fast and Simple Method for Detecting Identity-by-Descent Segments in Large-Scale Data. Am J Hum Genet 2020;106(4):426-37.
6. Lohmann K, Redin C, Tonnies H, et al. Complex and Dynamic Chromosomal Rearrangements in a Family With Seemingly Non-Mendelian Inheritance of Dopa-Responsive Dystonia. JAMA Neurol 2017;74(7):806-12.
